# Predicting the trend of SARS-CoV-2 mutation frequencies using historical data

**DOI:** 10.1093/bioinformatics/btaf508

**Published:** 2025-09-17

**Authors:** Xinyu Zhou, Yi Yan, Kevin Hu, Haixu Tang, Yijie Wang, Lu Wang, Chi Zhang, Sha Cao

**Affiliations:** Center for Computational Biology and Bioinformatics, Department of Medical and Molecular Genetics, School of Medicine, Indiana University, Indianapolis, IN, 46202, United States; Department of Computer Science, Indiana University Bloomington, Bloomington, IN, 47408, United States; Division of Microbiology Devices (DMD), Office of In Vitro Diagnostics and Radiological Health (OHT7), Center for Devices and Radiological Health (CDRH), U.S. Food and Drug Administration, Silver Spring, MD, 20993, United States; Center for Computational Biology and Bioinformatics, Department of Medical and Molecular Genetics, School of Medicine, Indiana University, Indianapolis, IN, 46202, United States; Carmel High School, Carmel, IN, 46032, United States; Department of Computer Science, Indiana University Bloomington, Bloomington, IN, 47408, United States; Department of Computer Science, Indiana University Bloomington, Bloomington, IN, 47408, United States; Department of Information Systems and Operations Management, Ball State University, Muncie, IN, 47306, United States; Center for Computational Biology and Bioinformatics, Department of Medical and Molecular Genetics, School of Medicine, Indiana University, Indianapolis, IN, 46202, United States; Department of Biomedical Engineering, Oregon Health and Science University, Portland, OR, 97201, United States; Center for Computational Biology and Bioinformatics, Department of Medical and Molecular Genetics, School of Medicine, Indiana University, Indianapolis, IN, 46202, United States; Department of Biomedical Engineering, Oregon Health and Science University, Portland, OR, 97201, United States; Department of Biostatistics and Health Data Science, School of Medicine, Indiana University, Indianapolis, IN, 46202, United States

## Abstract

**Motivation:**

As the SARS-CoV-2 virus rapidly evolves, predicting the trajectory of viral mutations has become a critical yet complex task. A deep understanding of future mutation patterns, in particular the mutations that will prevail in the near future, is vital in steering diagnostics, therapeutics, and vaccine strategies for disease control.

**Results:**

In this study, we developed a model to forecast future SARS-CoV-2 mutation surges in real-time, using historical mutation frequency data from the USA. We transformed the temporal prediction problem into a supervised learning framework using a sliding window approach. This involved breaking the time series of mutation frequencies into very short segments. Considering the time-dependent nature of the data, we focused on modeling the first-order derivative of the mutation frequency. We predicted the final derivative in each segment based on the preceding derivatives, employing various machine learning methods, including random forest, XGBoost, support vector machine, and neural network models. Empowered by the novel transformation strategy and the high capacity of machine learning models, we observed low prediction error that is confined within 0.1% and 1% when making predictions of mutation rates for the future 30 and 80 days, respectively. In addition, the method also led to a notable increase in prediction accuracy compared to traditional time-series models, as evidenced by much lower MAE (Mean Absolute Error) and MSE (Mean Squared Error) for predictions made within different time horizons. To further assess the method’s effectiveness and robustness in predicting mutation patterns for unforeseen mutations, we first designed a synthetic case where we categorized all mutations into three major patterns. The model demonstrated its robustness by accurately predicting unseen mutation patterns when training on data from two pattern categories while testing on the third pattern category, showcasing its potential in forecasting a variety of mutation trajectories. We then applied our method to prediction for a recent time frame between 1 January 2025 and 10 June 2025, for both the USA and UK, where the model training was conducted using frequency sequence data collected between 12 December 2019 and 26 January 2023 in the USA. The model demonstrated superior performance for both datasets.

**Availability and implementation:**

To enhance accessibility and utility, we built our methodology into a GitHub package (https://github.com/ZhouXY199502/SWD). Our method has the potential applicability to study other infectious diseases or forecasting tasks, thus extending its relevance beyond the current COVID pandemic.

## 1 Introduction

The COVID-19 pandemic, caused by the severe acute respiratory syndrome coronavirus 2 (SARS-CoV-2), continues to pose significant global challenges. As of July 2025, over 778 million people have been infected, resulting in the tragic loss of around seven million lives (World Health Organization 2023). These numbers include only confirmed, laboratory-verified cases reported through WHO surveillance channels, and the actual numbers are likely much higher. A pivotal aspect of the pandemic has been the emergence of numerous SARS-CoV-2 variants with different properties such as increased transmissibility, changes in disease severity, and varied responsiveness to vaccines and treatments (Hacisuleyman *et al.* 2021, Tao *et al.* 2021, Telenti *et al.* 2022, Carabelli *et al.* 2023), significantly affecting the effectiveness of public health interventions (Harvey *et al.* 2021, Walensky *et al.* 2021, Telenti *et al.* 2022, Carabelli *et al.* 2023). Hence, understanding the mutation dynamics of the virus is critical in navigating the ongoing crisis efficiently (Chen *et al.* 2022). Mutations in the viral genome occur due to errors in RNA replication processes, resulting in variations such as base substitutions, insertions, and deletions (Pachetti *et al.* 2020,  Thakur *et al*. 2022). With ongoing transmission, these microscopic changes can be replicated in subsequent rounds of infection and potentially alter the virus’s characteristics, further influencing the efficacy of diagnostic tools, vaccines, and therapies (Pachetti *et al*. 2020, Thakur *et al*. 2022).

To better understand the virus mutation dynamics, the global scientific community has collected a substantial amount of genomic data, with about 16 million SARS-CoV-2 sequences currently available through the Global Initiative on Sharing All Influenza Data (GISAID) (Gangavarapu *et al.* 2023). This extensive database has catalyzed a new age in viral genomic research, fostering near real-time surveillance of the pandemic and profoundly influencing public health policies (Oude Munnink *et al.* 2021, Gangavarapu *et al.* 2023). For instance, researchers have developed real-time monitoring systems to track the emergence and spread of new variants globally (Singer *et al.* 2020, Chen *et al.* 2021, World Health Organization 2023). Researchers have also relied on the mutation fingerprints for studying real-time molecular epidemiology (Candido *et al.* 2020, Lu *et al.* 2020, Du Plessis *et al.* 2021, Volz *et al.* 2021). Several recent reports describe new methods for utilizing machine learning and computational biology to predict potential future mutations and their implications, and forecast the potential impact of mutations on the trajectory of the pandemic (Maher *et al.* 2022, Najar *et al.* 2023). The differentiation of the SARS-CoV-2 virus according to geographic locations (Khalid *et al.* 2023) and racial differences, as well as the impacts it has on humans post-infection (Kim *et al.* 2023), also constitute hotspots in research.

Despite the breadth of ongoing scientific investigation, a critical challenge remains to the accurate forecasting of dominant mutations in the imminent future. Presently, a myriad of forecasts exists concerning SARS-CoV-2 variants (Bai *et al.* 2021, Zahradník *et al.* 2021, Ullah *et al.* 2022, Popovic 2022). Although insightful, these methods may fall short of predicting variants that emerge in the future because of the complexity of host–virus interaction and the plasticity of virus biology (Jacobs *et al.* 2023). In particular, the current methods primarily focus on predicting macro-level variants, potentially overlooking the subtleties of individual mutations that significantly impact the virus’s behavior. Additionally, these methods do not provide a real-time application, which is crucial for public health responses.

While variants have gained significant attention, it is their constituent mutations that directly influence the effectiveness of diagnostic tools and vaccines. This raises a compelling proposition: rather than focusing solely on predicting dominant variants, we may shift our attention to the underlying mutations. In this study, we aim to predict the virus genomic dynamics by focusing on individual mutations. We propose to utilize historical mutation frequency data from US patients in GISAID to forecast future mutation frequency patterns in real-time, while also determining the maximum time horizon within which mutation frequencies can be accurately predicted with minimal deviation. Recognizing the limitations of traditional time series-based models, we have devised a data transformation strategy, thereby converting a time series modeling challenge into a supervised learning task. This innovation enables the implementation of advanced machine learning methods to harness their critical predictive capacities. Our research offers several key contributions: (i) Studying the virus genomic dynamics at the unit of individual mutations, an approach not previously pursued. (ii) A novel data transformation method designed to harness machine learning capabilities for mutation frequency predictions. (iii) Modeling based on the first-order derivative of mutation frequency, enhancing prediction accuracy and capturing temporal relationships. (iv) The establishment of evaluation metrics for various methods, enabling a comprehensive assessment of their effectiveness that could be directly transplanted to the study of other infectious diseases.

Throughout this article, we employ the term “mutation” to denote base-level changes compared to the Wuhan-Hu-1 reference sequence (GenBank accession: NC_045512.2).

## 2 Results

### 2.1 Problem formulation

Assume that for the SARS-CoV-2 viral genome, there are in total *K* genomic mutations whose daily occurrence frequencies have been observed from day 1 to *N* in a certain population. For a specific mutation *k*, we denote the mutation frequency for day *i* as $f_{ki}$, where i=1,…,N;k=1,…,K. Our objective is to learn a forecasting function *g* that can accurately predict the mutation frequency $f_{k,N+1},\ldots ,f_{k,N+T}$ for a specific mutation *k* on future *T* days, based on the historical rates observed from day 1 to day *N*. Formally, we define the function as:


(1) $$g:F_{O}^{\left(K\times N\right)}\to F_{E}^{\left(K\times T\right)}$$

**Figure 1.**
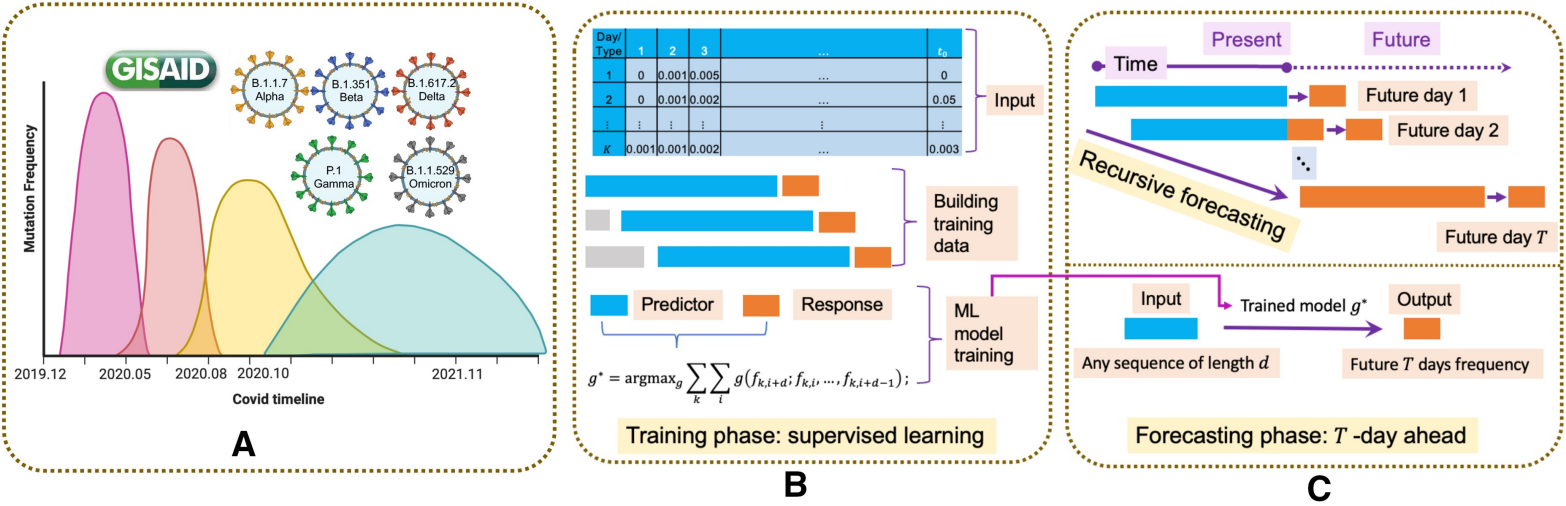
Schematic outline of our method. (A) Different COVID variants have appeared so far, each with different combinations of mutation types with varied mutation frequencies observed in the population. To forecast the mutation frequency changes, our prediction model consists of a training phase (B) and forecasting phase (C). (B) In the training phase, for all mutation, the historical mutation frequency for all mutation types is calculated and provided as tabular input, and using a sliding window approach, we could apply machine learning model to predict the last mutation pattern using preceding ones. (C) In the forecasting phase, future mutation frequencies is predicted day by day with a recursive fashion.

This function takes as input the matrix of observed mutation frequencies $F_{O}\in R^{K\times N}$, where each element represents the frequency of mutation *k* on day *i*, and outputs a matrix $F_{E}\in R^{K\times T}$, with each row corresponding to the predicted rate of mutation for each of the *K* types for the future *T* days (see Fig. 1). The challenge is to design and train *g* such that it minimizes a loss function $L(F_{E}^{\left(K\times T\right)},F_{\text{true}}^{\left(K\times T\right)})$, where $F_{E}^{\left(K\times T\right)}=g(F_{O}^{\left(K\times N\right)})$, and $F_{\text{true}}^{\left(K\times T\right)}$ is the underlying truth. This loss function quantifies the accuracy of the predictions, and the goal is to find the right *g* that could minimize this loss over the training dataset. Note that, depending on the choice of the model, *g* may vary across different *k*, such as a time series model. This mathematical formulation sets a clear objective for the learning task, focusing on predicting future mutation frequencies based on historical data. Clearly, there are three key aspects with this formulation: constructing the training and testing dataset; choice of family of prediction function *g* and the evaluation metrics. In Sections 2.2–2.4, we will expand on how we approached this forecasting task from the aforementioned three aspects.

### 2.2 Harnessing the power of machine learning in predicting mutation frequencies

As described in Section 2.1, our task centers on temporal forecasting. While traditional time-series models and curve-fitting techniques like spline methods are common choices, they may struggle with the complex, hard-to-learn patterns of mutation frequency. As we’ll demonstrate, these methods do not always yield the best results in our context. Nowadays, machine learning has become increasingly effective in deciphering complex biological patterns. Leveraging this, we introduce an innovative approach: transforming each full-length temporal mutation frequency sequence into shorter, consecutive segments. This method, known as sliding window dissection (**SWD**), redefines a time series problem as a supervised prediction challenge. With SWD, each segment’s last data point becomes the response variable, while the preceding points serve as predictors. This setup integrates temporal dependencies into the supervised learning framework. It allows for more sophisticated modeling of nonlinear relationships and interactions using advanced machine learning techniques. Models like random forest, neural networks, support vector machines (SVM), and XGBoost are adept at learning intricate patterns and adapting to evolving dynamics. Most importantly, a key advantage of this transformation strategy is its ability to integrate mutation frequency data from different genomic mutation types into a single predictive model. This holistic approach could enhance the accuracy and robustness of our predictions, offering a more comprehensive understanding of the virus’s evolution.

Training the aforementioned advanced models is a pivotal step, highly dependent on the training dataset, which is constructed from the dynamic mutation observations of all observed genomic sites over time for all collected virus sequences of all different variants from GISAID (Fig. 1A). From GISAID, we collected the mutation frequency data spanning a total of 1130 days (12 December 2019 to 26 January 2023), for a total of 87 mutation types as our main training and testing cohort, shown as the tabular input of Fig. 1B. A mutation type is a mutation event that is marked by the location in the whole genomic sequence where a particular change occurred. Details on the 87 mutation types/events, as well as necessary pre-processing such as smoothing, are illustrated in Section 4. Fig. 1B illustrates how we constructed our training data using a sliding window approach, where each blue and orange segment denotes one set of observations for predictor and response, respectively. In this method, for a segment of length d+1 within the range [1,…,N], specifically [i,…,i+d], we treat $f_{k,i},f_{k,i+1},\ldots ,f_{k,i+d-1}$ as predictors (denoted as $x_{ki}$, blue segment in Fig. 1B) and $f_{k,i+d}$ as the response (denoted as $y_{ki}$, orange cell in Fig. 1B), where *d* is the size of the sliding window. By iterating over all values of *k* and *i*, we obtain all possible sliding segments $\left(x_{ki},y_{ki}\right),k=1,\ldots ,K,i=1,\ldots ,N-d$. These segments form our training data, helping us identify the most effective prediction model. The remaining segments are used as testing data to evaluate the methods’ performance. This method prepares our data for application in various machine learning models. Each model can be trained using the training data and assessed using the testing data. It’s important to note that, due to the temporal nature of the data, all training data segments must precede the testing data segments in time. This ensures the validity and reliability of our forecasting approach.

Figure 1C illustrates the forecasting process for the mutation frequency of a specific mutation event over the next *T* days. The prediction begins with ${\hat{f}}_{N+1}$, using observed rates $\left\{f_{N-d+1},\ldots ,f_{N}\right\}$ and the trained model $g^{*}$ (Future day 1 in Fig. 1C). The process then uses $\left\{f_{N-d+2},\ldots ,f_{N},{\hat{f}}_{N+1}\right\}$ as input to recursively predicts ${\hat{f}}_{N+2}$ (Future day 2 in Fig. 1C), where ${\hat{f}}_{N+1}$ is obtained in the previous step. This recursive process continues until all future *T* days predictions are obtained (Future day *T* in Fig. 1C). A key advantage of our approach is the ability to combine mutation patterns from various types to create a unified prediction model. In contrast, while traditional time series models excel at capturing periodic or seasonal trends by training on individual sequences, they must train for each site individually.

In our data engineering process, we initially used a sliding window method to create $x_{ki}$. However, this method overlooked the sequence order and auto-correlation in $x_{ki}$, which is a temporal sequence consisting of $\left(f_{ki},f_{k,i+1},\ldots ,f_{k,i+d-1}\right)$. To address this, we shifted our focus from modeling daily mutation frequencies to their first-order derivatives. Consequently, our new predictor and response variables are $x_{ki}^{*}=\left(f_{ki}^{\prime },f_{k,i+1}^{\prime },\ldots ,f_{k,i+d-1}^{\prime }\right)$ and $y_{ki}^{*}=f_{k,i+d}^{\prime }$, where $f_{ki}^{\prime }$ represents the first-order derivative of the mutation frequency at day *i* for location *k*, approximated as $f_{ki}^{\prime }=\frac{f_{k,i+j}-f_{ki}}{j}$, where *j* is a parameter for step size. Details can be found in Section 4. This approach to first-order derivatives allows us to more effectively capture the dependencies within the predictor variables. Another significant benefit is the reduction of auto-correlation among predictors. This auto-correlation, arising from overlapping data points in consecutive sequences, can skew the learning process of the model and lead to biased, overly optimistic evaluations. By using first-order derivatives and introducing lag variables, we decrease the dependency between consecutive observations, enhancing the robustness and reliability of our modeling.

Then, on the transformed data, we explored a wide range of prediction techniques. These include SVM with linear (S${\;}_{L}$) and radial (S${\;}_{R}$) kernels, random forest (RF), XGBoost (XG), forward neural network (FNN), and linear regression (LR) as a straightforward and interpretable benchmark. This selection spans various modeling strategies to ensure a comprehensive analysis by blending linear and non-linear, parametric and non-parametric models, alongside ensemble methods and neural networks. We refer to these as supervised learning methods, denoted by SWD+X, where X represents one of S${\;}_{L}$, S${\;}_{R}$, RF, XG, FNN, and LR. In contrast, we also evaluated traditional methods that have been used to model time series data, such as ARIMA (ARI) representing the autoregressive integrated moving average method, Prophet (PRO), *k*-nearest neighbor (*k*NN), cubic-spline (C${\;}_{S}$), and B-spline (B${\;}_{S}$). ARIMA and Prophet are autoregressive models typically used in time series analysis, while *k*NN and spline methods are functional methods, designed to fit curves to time series data. For convenience, these were all referred to as time series learning methods. A key part of our analysis was comparing these traditional models against the supervised learning methods.

### 2.3 Evaluations on overall performances

This section provides a comprehensive evaluation of our proposed methods, focusing on their robustness to different hyperparameter settings and their performance relative to time-series approaches using Mean Squared Error (MSE) and Mean Absolute Error (MAE). These metrics assess the deviation between predicted and actual mutation frequencies in test data. For supervised learning, we split the whole time frame at a specific date, $t_{0}$. Here, we set $t_{0}=500$. Data before day $t_{0}$ served as training material, covering all mutation events. We then randomly selected 100 temporal points $t_{1}$, where $t_{1}>t_{0}$, and tested predictions for days 1, 7, 14, 30, 80 beyond each $t_{1}$ for each of the 87 mutation events, comparing them against the actual mutation frequencies. Time series methods also used $t_{0}$ as a dividing point. Here, the mutation frequency of each mutation event was learned independently, using data before $t_{0}$ for training. We then predicted mutation frequencies after any randomly selected $t_{1}$ and also compared them with the actual values using MSE and MAE metrics. MSE/MAE across all $t_{1}$ and all 87 mutation events was averaged to obtain the final error evaluations for each time horizon and each method under each setting. For more information, refer to Section 4. Importantly, $t_{0}$ was not necessarily set close to the end of the time series, which is at day 1130. This choice avoids cases where most of the mutation frequency curves become flat and near zero towards the end of the time frame, as seen in Fig. 1, available as supplementary data at *Bioinformatics* online, ensuring a more robust evaluation of the methods under varied mutation frequencies. Additionally, setting $t_{0}=500$ allows enough data points in the training set, to capture the variations in the data.

**Figure 2.**
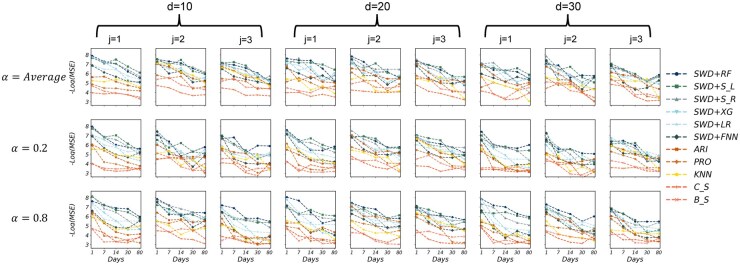
Performance evaluations of all 11 methods under different hyperparameter configurations. Each subplot shows the prediction performance over five future time horizons (1, 7, 14, 30, and 80 days), with the *x*-axis indicating the prediction horizon and the *y*-axis representing the negative log-transformed MSE. The three rows correspond to different smoothing approaches: simple average on the first row, EMA with α=0.2 on the second row, and EMA with α=0.8 on the last row. The columns are grouped by the sliding window sizes *d *= 10, 20, and 30, and derivative step size *j *= 1, 2, and 3. The supervised learning methods are in cold colors, while the time series methods in warm colors.

In assessing our methods’ sensitivity to different hyperparameter settings, we are concerned with three hyperparameters: smoothing approach (average, EMA α=0.2 and EMA α=0.8), sliding window size d=10,20,30 and first derivative step size j=1,2,3. See Section 4 for more details on the hyperparameters. Figure 2 shows the performance of the six machine learning methods under different hyperparameter settings, comparing to time series methods. Each subplot reports the negative logarithm of the mean-squared error (−log MSE) at five forecasting horizons (1, 7, 14, 30 and 80 days); larger values therefore indicate higher predictive accuracy. Curves in cold colors represent the SWD-based supervised methods, whereas warm colors denote time-series approaches. The sliding window parameter d=10 and d=20 deliver highly similar performances, whereas d=30 markedly reduces accuracy. Within each *d*, we explore the different derivative step size, and observed that setting j=1 yields consistently superior performances. When looking at the impact of the smoothing approaches, we noticed that setting α=0.8, which places greater weight on recent observations, demonstrates a modest but consistent improvement over the α=0.2 setting, while performing similarly to the simple average approach. Across all settings, the SWD methods showed relatively robust performances and almost always outperformed the time series methods. Hence, in our later experiments, we fixed the use of average smoothing, and set d=20,j=1 by default.

Table 1 provides a detailed numerical comparison of the 11 methods’ performance. The respective average MAEs and MSEs between the predicted and true mutation frequencies for predicting future day 1, 7, 14, 30 and 80 (shown as rows) for the six supervised learning methods and five time series methods (shown as columns) are shown. Here, MAE and MSE are averaged across 100 random temporal locations (beyond $t_{0}=500$) and all 87 mutation events. The cells denote performances of supervised learning methods and time series methods, respectively. Again, the supervised learning methods demonstrate substantially lower MAE and MSE. Among the supervised learning methods, SWD+RF showed notable performances compared to others, followed by SWD+S_R and SWD+S_L. In the table, for each scenario, the lowest MAE/MSE is highlighted in bold.

**Table 1.** Evaluating different learning approaches in forecasting mutation frequency for the future 1, 7, 14, 30, and 80 days.

	Machine learning						Time series				
Methods	SWD+RF	SWD+S_L	SWD+S_R	SWD+XG	SWD+LR	SWD+FNN	ARI	PRO	KNN	C_S	B_S
MAE (day 1)	2.03e−05	2.15e−05	**1.80e−05**	2.87e−05	2.72e−05	2.60e−05	2.20e−04	2.19e−03	5.09e−04	5.68e−03	4.78e−03
MAE (day 7)	**3.24e−05**	4.91e−05	4.04e−05	8.94e−05	6.40e−05	5.89e−05	3.11e−04	2.89e−03	3.99e−04	5.71e−03	4.70e−03
MAE (day 14)	**5.05e−05**	8.85e−05	8.59e−05	2.25e−04	1.04e−04	9.40e−05	2.16e−04	3.86e−03	2.92e−04	5.68e−03	4.48e−03
MAE (day 30)	**9.83e−05**	1.98e−04	2.61e−04	6.92e−04	1.78e−04	1.55e−04	1.15e−04	4.07e−03	3.31e−04	5.32e−03	4.85e−03
MAE (day 80)	**3.45e−04**	6.06e−04	1.27e−03	2.60e−03	5.55e−04	5.88e−04	4.72e−04	3.32e−03	3.98e−04	2.99e−03	1.23e−02
MSE (day 1)	**1.41e−08**	1.73e−08	1.45e−08	1.51e−08	4.28e−08	4.22e−08	1.03e−05	3.35e−05	1.16e−05	1.16e−04	4.78e−03
MSE (day 7)	1.95e−08	3.42e−08	**1.50e−08**	2.21e−08	2.40e−07	2.40e−07	6.63e−06	4.78e−05	8.87e−06	1.15e−04	9.30e−05
MSE (day 14)	3.61e−08	5.41e−08	**2.84e−08**	1.62e−07	5.53e−07	5.60e−07	2.81e−06	7.04e−05	7.86e−06	1.11e−04	9.80e−05
MSE (day 30)	1.45e−07	**1.30e−07**	1.89e−07	1.44e−06	1.08e−06	1.10e−06	1.99e−07	7.10e−05	1.21e−06	9.25e−05	1.20e−04
MSE (day 80)	2.29e−06	**9.83e−07**	4.02e−06	2.29e−05	3.90e−06	3.91e−06	9.89e−06	5.60e−05	1.87e−06	5.67e−05	6.22e−04

The bold values denote the method with the highest accuracy.

Notably, linear regression, under our data transformation framework, outperforms all time series methods in predicting future days 1, 7, 14, and 80, and sometimes even excels XGBoost and FNN. In the time series category, none can match supervised learning methods for shorter predictions (future days 1, 7, 14). For longer forecasts (future days 30, 80), ARIMA, the best among all selected time series learning methods, starts to show comparable or slightly superior performance to linear regression, XGBoost, and FNN, but its performance is still much worse than random forest and SVM. We noticed that with increasing prediction time horizons, supervised learning methods face escalating errors, which is expected as a result of error accumulation during the recursive prediction phase (see Fig. 1).

**Figure 3.**
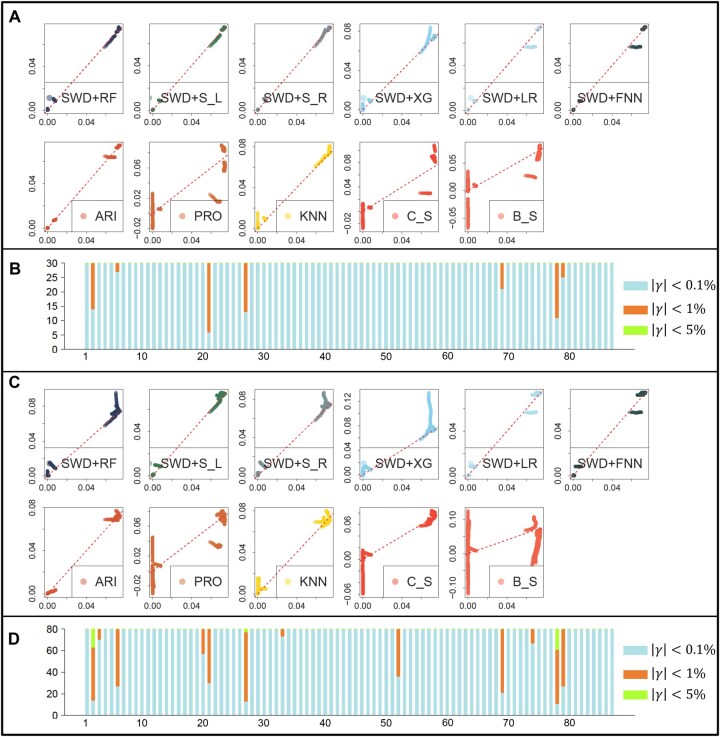
Comparisons of different learning methods in terms of prediction error for the future 30 and 80 days. (A) Scatter plot of predicted (*x*-axis) and observed (*y*-axis) mutation frequency for the next 30 days beyond $t_{0}=500$. Here, the dots pooled all 87 genomic mutations and all 30 days. (B) Barplot showcases the number of instances where the absolute difference between observed and predicted values is less than 0.1% (blue), 1% (orange), and 5% (green), among the 30-day prediction horizon beyond $t_{0}=500$ using SWD+RF. Here, *x*-axis denotes the 87 genomic mutations, and *y*-axis denotes the number of incidences of different error levels. (C) Scatter plot for predicting future 80 days similar to A. (D) Bar plot of level of deviations in predicting future 80 days similar to B.

Next, we compare the supervised learning and time series learning methods by taking a more refined look at the prediction accuracy. Figure 3 offers an in-depth look at each method’s performance. Similar to Fig. 2 and Table 1, by training with mutation frequency data up to day $t_{0}=500$, we predicted mutation frequencies for the subsequent 30 or 80 days for all 87 mutation types and compared these predictions with actual data using scatter plots and barplots. In Fig. 3A and 3C, scatter plots of observed versus predicted mutation frequencies, by pooling data from all 87 genomic mutations, reveal the extent of deviation for each method. Supervised learning methods are represented in cold colors, while time series methods are in warm colors. Among supervised methods, random forest (SWD+RF) and SVM (SWD+S${\;}_{L}$ and SWD+S${\;}_{R}$) demonstrate notable effectiveness, as evidenced by the overall placements of the points on the x=y axis. Interestingly, linear regression (SWD+LR) also shows commendable performance, surpassing time series methods, and provides comparable performance to XGBoost (SWD+XG), and forward neural network (SWD+FNN). Figure 3B and 3D quantify the deviation between predictions and observations for each of the 87 genomic locations from a discretized perspective. We assess how often predictions vary from actual values by 0.1%, 1%, and 5% when making predictions for the future 30 and 80 days, using one of the best-performing method, SWD+RF. This visual analysis indicates that for most mutations, the prediction error for the upcoming 30 days is under 0.1%, and never exceed 1%. Even for the 80-day forecast, deviations exceeding 1% are rare and always within 5%. The results highlight the reliability of 30-day predictions, especially with RF and SVM, as evidenced by scatter points closely aligning with the x=y axis. The majority of mutations show deviations below 0.1%, and all stay under 1%. For 80-day forecasts, despite higher deviations seen in the scatter plot (Fig. 3C), linear kernel SVM emerges as the most accurate, and random forest also performed comparably well, with almost all deviations remaining below 1% as shown in Fig. 3D.

### 2.4 Evaluations on capturing of the mutation frequency dynamics

**Figure 4.**
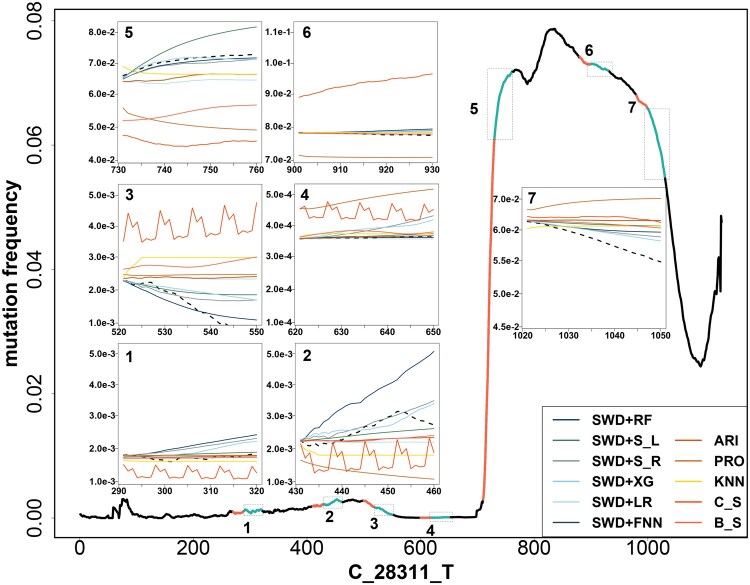
Prediction performance on seven 30-day future segments of “C_28311_T.” Here on the smoothed mutation frequency sequence of “C_28311_T” (black line) collected between 12 December 2019 and 26 January 2023, we selected seven distinct temporal locations, each comprising a 20-day input segment (marked in red) used for forecasting, followed by a 30-day ground truth segment (marked in cyan) used for evaluation. For each cyan segment, predictions from 11 different algorithms are shown. For each cyan segment, we demonstrated the prediction performance of the 11 different algorithms on predicting future 30 days. In each panel figure, the *x*-axis denotes the days, and *y*-axis the predicted or true mutation frequency.

The mutation frequency curves often exhibit numerous fluctuations, characterized by abrupt increases and declines, indicative of the unpredictable nature of viral mutation patterns (see Fig. 1, available as supplementary data at *Bioinformatics* online). To assess the capability of the proposed methods in managing such irregularities, we selected two specific mutation events, namely “C_28311_T” and “AG_28878_TC.” These two particular mutations are marked by a considerable number of segments exhibiting high fluctuations. For both mutations, we further handpicked seven segments that represent the fluctuations that spread throughout the whole time frame. We then evaluate how different learning approaches could handle the fluctuating mutation frequencies. For “C_28311_T,” shown in Fig. 4, the bold curve represents its daily mutation frequency, and the curve is marked mostly black, with seven red segments, and seven cyan segments each right after one red segment. For each supervised learning method, its model parameter is first trained using all the mutation segments that arose from other mutation types, excluding “C_28311_T.” To make prediction, the red segment, which is of length d,d=20, serves as input predictors to predict the next day 1, day 2,…, until day 30 in a recursive fashion, i.e., the cyan segment; for time series methods, to make predictions for each cyan segment, all the data points preceding this segment is used as training data to solve the model parameters, and prediction is directly made for the days within the cyan segment. We demonstrated the prediction of the future 30 days here, as this is a reasonable time frame that could allow sufficient time to alert the public and health policymakers to take necessary actions. Note the limitation with the time series model, which could not absorb information from other mutation types during the training phase. The seven-panel figures showcased the comparisons of predictions made by different learning methods with the true mutation frequencies, each for one handpicked segment, where the true mutation frequency is marked by a black-dashed line. The supervised learning approaches (solid segments of cold colors) exhibited superior predictive performance relative to the time series learning approaches (solid segments of warm colors), as evidenced by their overall closeness to the observations (black dash segments).

Specifically, when the segments are experiencing steep increase or decrease but with different rates (segments 5, 6, 7), machine learning methods that directly model the first derivative demonstrated a robust ability to track these dynamic shifts. Conversely, time series methods generally struggled to adjust to the changing slopes. In instances of minor to moderate overall changes in slope but with certain fluctuations (segments 1, 2, and 3), supervised learning algorithms adeptly captured these fluctuations, whereas time series models often failed to reflect these changes accurately. When the segments showed minimal variation (segment 4), most methods performed similarly well, although supervised learning algorithms typically offered predictions that more closely approximated the actual observations. Among the supervised learning techniques evaluated, the two Support Vector Machine (SVM) models consistently outperformed others by effectively capturing fluctuations. We could draw similar conclusions for mutation “AG_28878_TC” in Fig. 2, available as supplementary data at *Bioinformatics* online.

In summary, while time-series models are effective in many forecasting situations, their ability to predict the evolving dynamics of mutations is limited. We identify three key reasons for this: (i) Time-series models excel at identifying consistent seasonal trends, which mutation patterns typically lack; (ii) These models often train on each mutation independently, missing out on valuable inter-mutation information; (iii) Time-series models generally face difficulties in making accurate long-term predictions in dynamic contexts such as mutation evolution. These limitations highlight the necessity for more sophisticated modeling techniques in complex forecasting scenarios, a point also emphasized by Coccia (2023). In contrast, our machine learning based methods demonstrate greater flexibility and applicability across different mutation types. It effectively captures the non-linear and intricate patterns hidden within mutation trajectories, offering more reliable predictions in these complex biological systems.

### 2.5 Evaluations of robustness in predicting unforeseen mutation patterns

Given the dynamic nature of viral evolution, a critical challenge for any forecasting model is its ability to accurately predict future rates for mutation types not previously encountered during its training phase. This challenge arises for two main reasons: first, novel mutations may emerge with historical mutation frequencies that were negligible and only recently became significant. Consequently, these mutations might not be represented in the training dataset, or the available data could be excessively noisy. Nonetheless, understanding how the mutation frequency of these emergent types will change is crucial. Second, while it would be ideal for a model to be exposed to all mutation types during training, a robust forecasting model must effectively capture the essence of viral evolution, even when some mutation types are absent from its training data. To evaluate the robustness of our SWD methods in making predictions for unforeseen mutations, we took two different routes. First, we devised a synthetic experiment where we categorized 87 mutation types into three primary categories. We then trained prediction models on two of these categories and tested them on the third, which had not been previously seen by the model. This approach simulates a realistic scenario of forecasting unseen mutations and assesses the model’s robustness and generalizability in predicting the evolution of novel viral mutations. Second, we collected the most recent daily mutation frequency data from both the USA and UK, spanning 1 January 2025 to 10 June 2025, and evaluated how well a model trained solely on historical USA data from 12 December 2019 to 26 January 2023 could forecast mutation trends in these more recent datasets.

**Figure 5.**
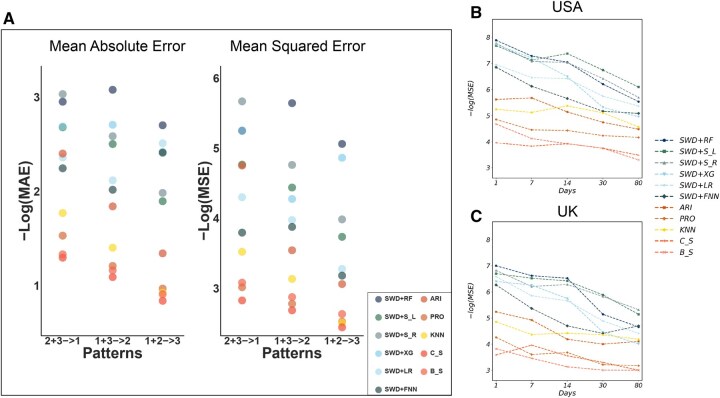
Robustness analysis. (A) The prediction accuracy (*y*-axis) measured by negative logarithm MAE (left) and MSE (right) for predicting future 30 days under three different scenarios (*x*-axis). For instance, (2 + 3 → 1) implies using pattern 2 and pattern 3 as the training set and pattern 1 as the test set. Similarly, cold and warm color bubbles indicate supervised and time series learning methods, respectively. (B) Performance comparisons for predicting future 1, 7, 14, 30, and 80 days mutation frequencies of the USA in a more recent time frame from year 2025. (C) Performance comparisons for predicting future 1, 7, 14, 30, and 80 days mutation frequencies of the UK in a recent time frame from year 2025.

**Figure 6.**
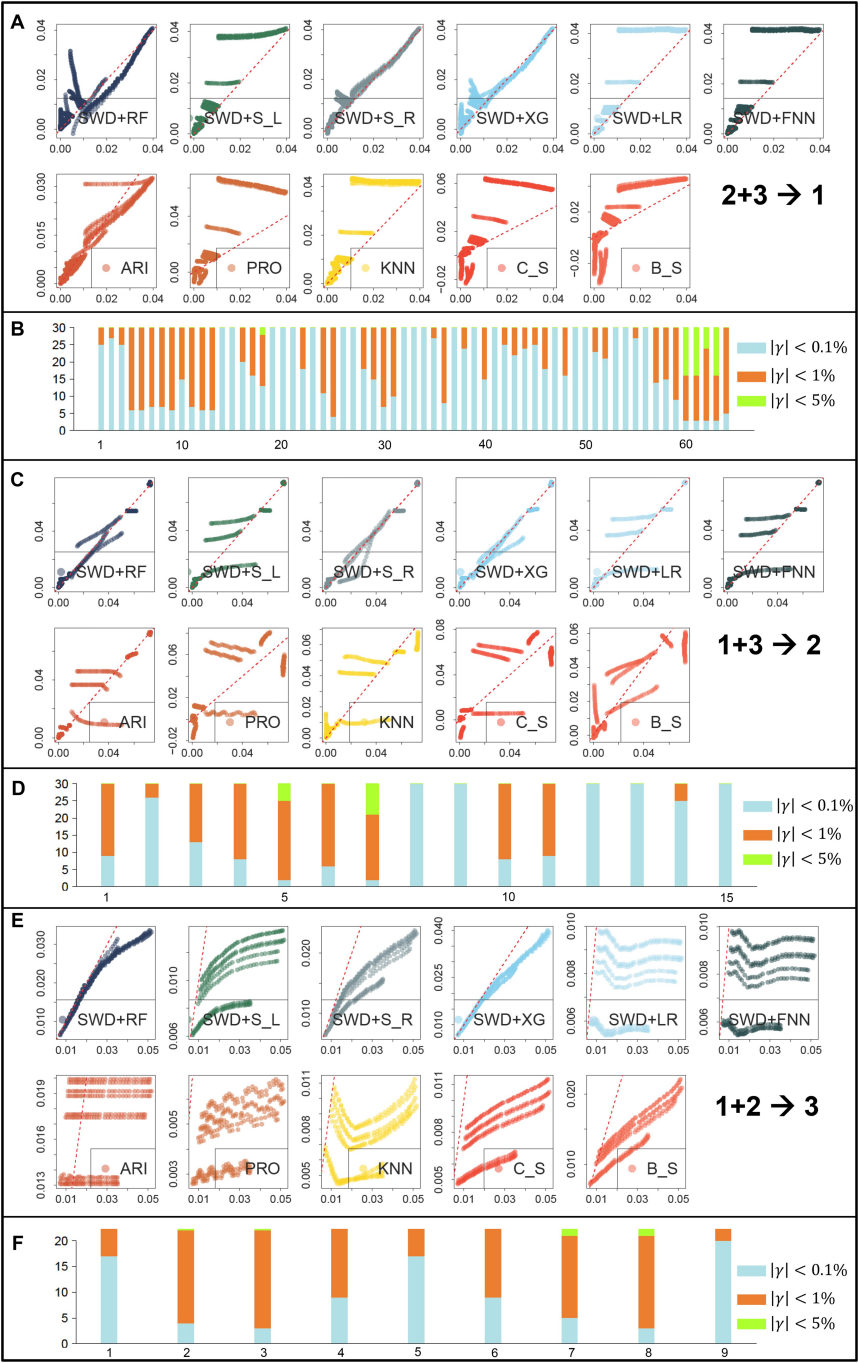
Robustness analysis. (A) Scatter plot of predicted (*x*-axis) and observed (*y*-axis) mutation frequency for the next 30 days when using pattern 1 and pattern 2 segments as the training data to predict mutation trend in pattern 3. Here, the dots pooled all genomic mutations from category 3 and all 30 days. (B) Barplot of different levels of deviations between predicted and observed mutation frequency for the next 30 days. (C) and (D) are similar to (A) and (B), but using pattern 1 and 3 to predict pattern 2, similarly for (E) and (F) which uses pattern 1 and 2 to predict pattern 3.

In the synthetic experiment, we have categorized the 87 genomic mutations into three distinct groups based on their mutation patterns. These groups are labeled as category 1, 2, and 3, comprising 63, 15, and 9 mutations, respectively (see Fig. 1, available as supplementary data at *Bioinformatics* online). Each category exhibits unique characteristics. Category 1 consists of mutation types where the highest mutation frequency remains below 5% (orange-red curves in Fig. 1, available as supplementary data at *Bioinformatics* online). Category 2 includes mutation types that peak earliest in the timeline, with a peak mutation frequency surpassing 5% (cyan curves in Fig. 1, available as supplementary data at *Bioinformatics* online). Category 3 features mutation types that exhibit their highest frequencies later in the timeline, with peak frequencies also above 5% (maroon red curves in Fig. 1, available as supplementary data at *Bioinformatics* online). Note that, here, we categorized the mutations based on their peak time and frequency to mimic the stepwise rise of SARS-CoV-2 lineages. For example, Category 2 groups the persistent, low-amplitude mutations that never rise far above background. They may capture classic hitch-hikers that have circulated since the earliest COVID wave. Category 1 peaked around April 2021, and may capture Alpha-era signature changes that rose to above 50% global prevalence by spring 2021 before being eclipsed (Andre *et al.* 2023), while Category 3, peaking around September 2021, may collect the Delta-defining changes that surged to dominance by September 2021 (Shiehzadegan *et al.* 2021). We demonstrate the methods’ performance in terms of MSE and MAE in Fig. 5A, and in Fig. 6, we show scatter plots of predicted and observed mutation frequency values and barplots of deviance between prediction and observations similar to Fig. 3.

Specifically, for each supervised learning method, we trained each model using data from two of the three mutation categories and then tested its ability to predict the third. For instance, we used data from categories 2 and 3 to predict category 1 (2 + 3→ 1), categories 1 and 3 for category 2 (1 + 3→ 2), and categories 1 and 2 for category 3 (1 + 2→ 3). These represent the *x*-axis in Fig. 5A. Our prediction process was structured as follows: we selected a specific time point, i.e. $t_{0},t_{0}=500$, where only data collected before and on $t_{0}$ for any two categories serve as training sets, and aimed to predict frequencies of mutations from the third category for 30 day horizon $(t_{1}+1$ to $t_{1}+30$), where $t_{1}>t_{0}$. The training data, drawn from two categories, always preceded the prediction point $t_{0}$. This setting was applied consistently for predicting mutation frequencies in categories 1, 2, and 3. When making predictions for a category, 100 different temporal locations, i.e., $t_{1}$, for each mutation type from the category, were randomly selected, and the average MAE/MSE across all 100 $t_{1}$ and all mutation types in the category was calculated and reported.

Figure 5A shows the overall performance of different methods in terms of negative and logarithm MAE and MSE. Overall, supervised learning methods demonstrated more desirable performance. In predicting unseen mutation types in categories 2 (using categories 1 and 3) and 3 (using categories 1 and 2), supervised learning methods significantly outshine all time series methods, as indicated by much lower MAE and MSE values. However, when forecasting for category 1, ARIMA (ARI) performs better than linear regression (LR) and forward neural network (FNN) in terms of both MAE and MSE, though it still largely lags behind the other four supervised methods. Table 2 provides the numerical values of average MAE and MSE for each method in predicting the future 30 days similarly setup as Fig. 5A. The lowest value for each scenario is highlighted in bold.

**Table 2.** Evaluating the methods performance for predicting future 30-days under different scenarios).^a^

	Machine learning						Time series				
Methods	SWD+RF	SWD+S_L	SWD+S_R	SWD+XG	SWD+LR	SWD+FNN	ARI	PRO	KNN	C_S	B_S
MSE (2,3 → 1)	5.62e−06	1.36e−05	**1.35e−06**	2.80e−06	1.99e−05	5.06e−05	4.43e−06	1.92e−04	4.76e−05	1.87e−04	8.28e−05
MAE (2,3 → 1)	1.12e−03	1.66e−03	**5.87e−04**	1.03e−03	1.74e−03	1.80e−03	9.98e−04	5.88e−03	2.69e−03	6.35e−03	4.65e−03
MSE (1,3 → 2)	**2.27e−06**	2.89e−05	1.09e−05	2.65e−05	4.21e−05	4.19e−05	7.18e−05	3.31e−04	1.16e−04	2.59e−04	1.33e−04
MAE (1,3 → 2)	**8.37e−04**	2.50e−03	1.64e−03	9.86e−04	3.04e−03	3.04e−03	3.62e−03	1.23e−02	6.27e−03	1.02e−02	6.87e−03
MSE (1,2 → 3)	8.67e−06	1.46e−04	6.53e−05	**6.84e−06**	2.10e−04	2.08e−04	2.18e−04	5.95e−04	4.89e−04	4.53e−04	2.32e−04
MAE (1,2 → 3)	1.99e−03	1.01e−02	6.55e−03	**1.91e−03**	1.23e−03	1.23e−03	1.14e−02	2.14e−02	1.88e−02	1.82e−02	1.23e−02
MSE (USA)	2.08e−07	6.84e−07	**1.05e−07**	3.11e−06	6.83e−06	5.72e−06	3.61e−06	1.12e−05	7.72e−06	5.21e−05	3.26e−04
MSE (UK)	**4.24e−06**	5.32e−06	6.79e−06	9.80e−06	1.58e−05	7.68e−06	2.21e−05	5.33e−05	1.23e−05	4.48e−05	4.77e−04

a The average MSE or MAE for each method (shown as columns) are presented under different synthetic and real-world scenarios (shown as rows). The top six rows are synthetic scenarios and the bottom two rows are for USA and UK data in a recent time frame. The scenarios resemble that of Fig. 5.

The bold values denote the method with the highest accuracy.

Figure 6 offers a visual inspection of the methods’ performance. Predicting unforeseen mutation patterns is inherently difficult, and this is reflected in the scatter plots where the predicted and true mutation frequencies do not always lie on the x=y axis (Fig. 6A, C, and E). Still, supervised methods, such as random forest (SWD+RF), SVM with a linear or radial kernel (SWD+S${\;}_{L}$ and SWD+S${\;}_{R}$), and XGBoost (SWD+XG), maintain desirable performance, with most predicted and observed values closely aligning with the x=y axis. Here, almost all time series methods, along with some supervised learning methods like SWD+LR and SWD+FNN, show scattered, random predictions, with predicted and observed data points diverging significantly from the x=y axis. The barplots in Fig. 6B, D, and F offer a direct interpretation of absolute prediction accuracy given by SWD+RF. While there are more instances of higher prediction deviations (indicated by orange and blue colors) compared to the overall model trained on all mutation types (as seen in Fig. 3), the majority of these deviations remain within 1%. Only in rare cases do the deviations exceed 1%, yet they still stay under 5%.

To further validate the robustness of our approach under real-world scenarios, we included two additional datasets from a recent time frame of 1 January 2025–10 June 2025, from USA and UK. We trained our model using data spanning 2019 to 2023 from USA, and tested whether the proposed methods can maintain their predictive performance for data collected in a time frame or region that it has never encountered. Figure 5B and C reported −log(MSE) for USA and UK, respectively, across forecasting horizons of 1, 7, 14, 30, and 80 days. Across both countries and all time horizons, our supervised learning methods consistently deliver lower prediction errors compared to the time series baselines, highlighting the robustness and strong generalization capability of our approach under real-world scenarios. Note that the MSE was again calculated based on averaging randomly selected 100 temporal locations and all of 87 genomic mutations for the USA and UK test data. The numerical values of the average MSE for predicting future 30-day periods was presented in the last two rows of Table 2 [MSE (USA) and MSE (UK)]. Comparing to Table 1, we saw a higher MAE and MSE in the synthetic and the recent USA/UK scenarios, which is expected. Training on the full model with all mutation sites provides stronger predictive power, while forecasting mutation dynamics more than two years into the future or across different geographic regions presents significant challenges. Nevertheless, our methods still achieved robust and desirable performance.

We have also analyzed the results when using raw frequency data instead of the first-order derivative data. Figure 3, available as supplementary data at *Bioinformatics* online provides a clear illustration of the advantages of our data transformation approach compared to directly modeling raw frequency data. When using the raw frequency data, which neglects the inherent temporal order and auto-correlation, the results are significantly worse. This is evident in the much more disorganized scatter plots shown in Fig. 3A, available as supplementary data at *Bioinformatics* online, particularly when predicting mutation frequencies for the upcoming 30 and 80 days. The issue becomes even more pronounced when performing the robustness analysis, which lacks any clear order or pattern. Such findings emphasize the crucial role of our data transformation strategy and the use of first-order derivatives in preserving the sequential nature of the data, both of which are key factors in achieving accurate and reliable predictions.

## 3 Discussion and conclusion

In this work, we developed an innovative model to forecast SARS-CoV-2 mutation surges by focusing on predicting the trajectories of individual genomic mutations and leveraging their historical frequency data. By transforming the prediction problem into a supervised learning framework, we employed a sliding window approach, specifically modeling the first-order derivative and employing advanced machine learning models. This approach yielded low prediction errors, and particularly, the errors were within 0.1% and 1% for forecasting mutation frequencies of future 30 and 80-days horizons, respectively, significantly outperforming traditional time-series models. The robustness of our model was further demonstrated by its high accuracy in predicting unseen mutation patterns, both in a synthetic setting and on a real data setting of US and UK mutation frequency data. On the contrary, traditional time-series models often struggle to capture the dynamic nature of these mutations (Chen *et al.* 2023).

Overall, our study suggests the feasibility of using historical frequency data to predict future surge patterns of mutation events, which is crucial in virology and epidemiology. Several key innovations exist. Firstly, while mutations at different genomic loci are not independent, our problem formulation of using the first derivative, instead of the original frequency, as predictors, could remove the strong autocorrelation that dominates individual series. Secondly, instead of fitting a separate temporal model to each mutation event, we transform every mutation-specific trajectory into sliding-window samples and pool those samples into one supervised-learning data set. The machine learners are therefore exposed simultaneously to the historical derivatives of all mutation events; during training, they can therefore discover cross-mutation covariation patterns that improve their global loss function. Thirdly, for learners such as random-forest, kernel-SVM, XGBoost, among others, their architectures automatically capture high-order interactions among predictors without explicit specification. The cross-mutation information is already embedded in the framework, meaning that dependencies are learned directly from the data and could be updated each day as new frequency data arrives.

One critical challenge of COVID surveillance is the monitoring of new variants. While our method operates at the individual genomic mutation, it continuously learns the trajectories of every observed mutation, and could potentially update the model each day as new data arrives. Hence, whenever a new variant emerges, one or more of its defining mutations will begin to rise, which are immediately captured as elevated first-order derivatives in the input window. As a result, the model will project higher future frequencies for those specific mutations. Real-time monitoring, in practice, depends on the cadence of model updates, which is itself constrained by the rate at which new data are collected and processed. Mutation frequencies of the population are often calculated at daily or even weekly resolution. Consequently, while model training may take several minutes to a few hours, depending on the size of the training data, it is much shorter than the new data update cycle, and hence will not hinder real-time monitoring. Retraining can occur daily or weekly to align with new frequency data availability. Once trained, the prediction step is computationally lightweight and can be executed in real time.

Nevertheless, our methodology has limitations. The varying correlations among mutations add complexity. Advanced feature engineering and selection methods might be necessary to address these inter-mutational correlations effectively. Transforming time-series data for supervised learning can lead to a loss of temporal information, possibly obscuring important temporal patterns and relationships. Integrating time-based features or developing hybrid models combining time-series and supervised learning approaches may enhance our methodology. In forecasting research, hybrid methods that combine classical time-series techniques with machine learning have been widely explored (Zhang 2003, Khashei and Bijari 2011, Sateesh Babu *et al.* 2016, Taylor and Letham 2018). The central idea is to leverage the nonlinear pattern-learning capacity of machine learning for the primary prediction, while utilizing time series models to capture any remaining temporal dependencies in the residuals. Advanced deep learning architectures such as Long Short-Term Memory (LSTM) networks and transformer-based models have shown strong performance in modeling complex temporal dependencies and nonlinearities in sequential data. These models can be trained directly on raw or feature-augmented mutation time series, and could potentially be combined with classical models through ensemble or multi-stage pipelines. For instance, stacked ensemble techniques, where multiple machine learning and time series models are combined via a meta-learner, may further improve predictive robustness by leveraging the strengths of diverse modeling paradigms (Wolpert 1992). Feature-level hybridization, where exogenous variables or state representations extracted from time series models are used as additional inputs for machine learning algorithms, also presents a flexible integration pathway (Masini *et al.* 2023). Moreover, recent neural hybrid frameworks such as N-BEATS and DeepAR, which combine deep neural architectures with autoregressive and attention mechanisms, represent powerful alternatives for sequence forecasting (Oreshkin *et al.* 2019, Salinas *et al.* 2020). Multi-task learning and transfer learning strategies may help address data scarcity and improve generalization across different mutation types or regions (Ruder 2017).

As future work, we may consider extending our existing SWD framework to hybrid statistical–machine learning models, with attention to two key considerations. First, SARS-CoV-2 mutation trajectories are highly irregular, spike-like, and largely nonseasonal; models designed for additive trend and seasonality have performed poorly in our setting, consistent with the lack of stable periodic structure. Second, hybrid models typically require per-series fitting and introduce additional hyperparameters, which would increase the operational complexity of daily or weekly model refreshes. Addressing these two challenges may bring practical improvements in long-horizon forecasting under shifting viral dynamics, especially during variant transitions, while maintaining scalability and responsiveness for real-time surveillance.

Notably, the versatility of our proposed methods extends beyond COVID-19, making them applicable to other diseases characterized by dynamic and intensively monitored mutation landscapes, as well as to a broad range of sequence data–based forecasting tasks. To facilitate broader use, we have encapsulated our approach into an easy-to-use R package. Using this package, researchers could perform data transformation, model training of a series of machine learning methods, as well as assessments of the methods’ performance using different metrics. The best-performing model can then be deployed for real-time prediction and continuously refined as new temporal data arrive.

## 4 Materials and Methods

### 4.1 Data collection

We obtained all of our mutation frequency data from the Global Initiative on Sharing All Influenza Data (GISAID) database. The GISAID database contains a large and rapidly growing collection of genomic data on the SARS-COV-2 from all over the world. We focused on the USA data only for several reasons: (i) The USA has experienced a diverse range of SARS-CoV-2 variants and has witnessed multiple waves of the pandemic, offering a rich and heterogeneous dataset that is reflective of the virus’s dynamic nature. (ii) The USA has a well-established infrastructure for genomic sequencing and reporting, contributing to the high quality and reliability of the available data. The extensive geographical and demographic diversity within the USA provides varied environmental, societal, and healthcare contexts, enabling a comprehensive exploration of the factors influencing viral mutation. (iii) The high incidence and prevalence of COVID-19 cases in the USA have resulted in a substantial volume of data, enhancing the statistical robustness of our analyses.

The GISAID database encompasses not only the genomic sequences of the virus but also associated metadata, providing details such as the location and date of sample collection. Leveraging this resource, we compiled an exhaustive record of virus mutation events spanning from 12 December 2019 to 26 January 2023. In essence, each variant sequence was aligned to a reference genome, enabling the identification of the type of mutation at specific nucleotide positions, for instance, G→T, along with the precise genomic location of the nucleotide. Additionally, information, including the geographic location (state and country) and the date of data collection for each patient’s virus, was also available. Each mutation event is designated based on the mutation type and its location; for example, “G_9053_T” signifies a substitution of base G to T at location 9053 in alignment with the reference genome. The historical data of each mutation event form the basis for predicting its future mutation frequency. Note that despite the genome size of the virus being 30 kb, most of the genomic locations have very low to zero mutation frequency, and eventually, we have included 300 genomic locations that have mutation frequency that peaked above 0.01 between 12 December 2019 and 26 January 2023. Among the 300 locations, many have mutation frequency curves that are identical to those of others. After further filtering, we have come down to 87 mutation types with unique mutation frequency curves. The method for calculating mutation frequency is detailed in Section 4.2.

In addition to the USA data collected between December 2019 and January 2023, which serves as our main training and testing data cohort, we have also collected frequency data of the 87 mutation types in a more recent time frame for the USA and UK, spanning 1 January 2025 to 10 June 2025. The raw sequence data and associated metadata were also downloaded from the GISAID database, and all processing steps follow the same procedure as the main USA data cohort detailed in Section 4.2.

### 4.2 Data preprocessing

To calculate the daily mutation frequency, we adopted a strategy akin to Mercatelli and Giorgi (2020). For any given day *i* and mutation *k*, we first identified the total count of samples collected during this time ($C_{i}$), and the subset of these individuals who carry the mutation *k* ($c_{ki}$). Thus, the daily mutation frequency was computed as $c_{ki}/C_{i}$. The raw daily mutation frequency data derived using this method exhibited a high degree of noise; hence, we implemented a smoothing step to mitigate the impact of transient spikes and drops in the data. Different smoothing approaches were explored: average and exponential moving average (EMA). For the average method, the smoothed daily mutation frequency is calculated as the average of day *i* and its preceding 9 days; for the EMA approach, denote $q_{ki}=c_{ki}/C_{i}$, and we calculate the smoothed frequency of mutation *k* at day *i*, i.e., $f_{ki}$, as $\alpha q_{ki}+(1-\alpha )f_{k,i-1}$. Here, α is a smoothing factor, where larger values of α have less smoothing effect and give greater weight to recent changes in the data, while values of α closer to 0 have a greater smoothing effect and are less responsive to recent changes. We explored two different α values, i.e., 0.2 and 0.8. This brings the total number of smoothing methods to 3: average, EMA with α=0.2 and EMA with α=0.8. The simple average smoothing is our default approach, which was shown to have better or similar performance compared to the EMA methods.

### 4.3 Data transformation with a sliding window approach

We employed a novel data transformation approach that makes it possible to use the powerful machine learning models. We assume that the mutation frequency follows certain Markov property, such that mutation frequency at day *i* depends on a window of *d* days that precede this date, i.e., $P(f_{ki}=f_{0}|f_{k,i-1},f_{k,i-2},\ldots ,f_{k1})=P(f_{ki}=f_{0}|f_{k,i-1},\ldots ,f_{k,i-d})$. Utilizing this property, we devise an approach to cut the whole time frame into overlapping time segments each of length d+1, and regard the last entry of the segment as response, and the preceding entries as predictors. Throughout this article, we treat d=20. We have also shown that the performance of the methods for *d* at different values, such as 10 and 30 in Fig. 2. A large number of segments could be obtained in such way, guaranteeing sufficient statistical power for the machine learning models. Training a machine learning model is to find a function $g^{*}$ such that $g^{*}={\text{argmin}}_{g}\sum_{k}\sum_{s\in S}L_{sk}^{g}$. Here S denotes the set of training time segments, which could be obtained from all or part of the mutation types or time frame, and $L_{sk}^{g}$ evaluates the loss for the *k*-th mutation type for one particular segment of length d+1, i.e., $s=(s_{1},\ldots ,s_{d+1})$ given by a function *g*, such as the squared difference between predicted and observed frequency for $f_{s_{d+1}}$.

Instead of looking for an optimal $g^{*}$ for modeling the raw mutation frequency, we turned to look for an optimal $g^{*}$ for minimizing the loss of the first derivative of the mutation frequency, which could better capture the temporal dependency of the frequency sequence. Here, for any date *i* and mutation type *k*, the first order derivative, $f_{ki}^{\prime }$, is approximated as the following: $f_{ki}^{\prime }=\frac{f_{k,i+j}-f_{ki}}{j}$. By default, we set j=1, but also showed the performance of the methods for *j* at different values, such as 2 and 3 in Fig. 2.

### 4.4 Constructing training and validation sets

To rigorously assess the performance of various methods, we partitioned the temporal mutation frequency data corresponding to 87 mutations into distinct training and testing datasets. This approach deviates from the traditional cross-validation technique, where training and testing instances are selected randomly. Instead, owing to the intrinsic temporal nature of our mutation frequency data, it is imperative to ensure that model training is confined exclusively to time frames prior to those for testing. This ensures that the model is not exposed to any mutation patterns from the testing data during training, maintaining the integrity and validity of our assessment. Specifically, for each of the 87 mutation, each with 1130 temporal observations, we obtained 1130-*d* overlapping temporal segments. For example, segment 1 contains mutation frequency data from date 1 through d+1, and segment 2 contains date 2 through d+2, and so on. Note that here, the frequency data is the smoothed daily frequency data. We set *d* to be 20.

The training and validation sets are constructed differently for different tasks. When measuring the overall performance (Figs 2 and 3, Fig. 3A, available as supplementary data at *Bioinformatics* online, Table 1), training data is obtained from all 87 mutations for segments that are prior to date $t_{0}$. Here $t_{0}=500$. And the testing is performed for time horizon $t_{1}+1,\ldots ,t_{1}+T$, for T=1,7,14,30,80 and $t_{1}>=t_{0}$. When measuring performance regarding individual genomic location, namely “C_28311_T” and “AG_28878_TC” (Fig. 4, Fig. 2, available as supplementary data at *Bioinformatics* online), training data is obtained for all other 86 mutation types, excluding the one that is being tested. Here, the time segments are also confined to date prior to $t_{0}=500$. Testing is performed for the seven hand-picked segments. When measuring performance regarding a large mutation type category (Figs 5A and 6, Fig. 3B, available as supplementary data at *Bioinformatics* online, Table 2), training is obtained as time segments from two categories prior to $t_{0}=500$, and testing is performed for the third category regarding the time horizon $t_{t}+1,\ldots ,t_{t}+T$, for $T=30,t_{1}>=t_{0}$.

### 4.5 Evaluation metrics

The MAE and MSE are the two main evaluation metrics. Unless otherwise specified, MAE and MSE are always calculated for multiple temporal locations across multiple mutation types. Usually, 100 temporal locations are randomly selected, and all or certain categories of the mutation types are pooled to obtain an average MAE or MSE.

### 4.6 Computational efficiency

Our method demonstrates strong scalability. With *d *= 20 and *j *= 1, assume we observed the frequency of 87 mutation types across 1000 days, which will yield roughly 85 000 training segments. Training the most robust but slowest method, SWD+RF, on the full dataset takes around 4.5 h. Using a random sampling of 75%, 50%, or 20% of the training segments reduces the training time to approximately 3, 2, and 1 h, respectively. For faster models with similarly strong performance, such as SWD+S_L and SWD+S_R, training on the full dataset completes within 2.5 h. These benchmarks were obtained using a laptop equipped with an Intel Core i7 processor, 32 GB of RAM, and a 1 TB SSD. Given that mutation frequencies are typically updated on a daily or weekly basis, the model training time, even at its maximum, is significantly shorter than the data update cycle, ensuring that real-time modeling update is not impeded. Once trained, the prediction process is computationally efficient and executes in real time.

### 4.7 Parameter settings for various machine learning and time series methods

- Random Forest (RF): the R library randomForest was utilized. The key default parameters include a number of trees (ntree) set to 500, mtry determined as the square root of the number of input variables, replace set to TRUE, and sampsize set to the sample size.
- SVM with Linear Kernel (S${\;}_{L}$): The SVM with a Linear Kernel was implemented using the e1071 library. The main default parameters include a misclassification cost (cost) of 1, gamma set to 1 divided by the data dimension, and epsilon set to 0.1.
- SVM with Radial Kernel (S${\;}_{R}$): The SVM with a radial Kernel was also implemented using the e1071 library. The main default parameters are: misclassification cost (cost) set to 1, gamma set to 1 divided by the data dimension, and epsilon set to 0.1.
- XGBoost (XG): The XGBoost model was developed using the R library xgboost. The objective was set to “reg: squarederror,” the learning rate (eta) was set to 0.3, the maximum depth was set to 6, the evaluation metric was set to “rmse,” and the number of training rounds was set to 300. The key default parameters include the booster set to “gbtree,” L2 regularization weight (lambda) set to 1, and L1 regularization weight (alpha) set to 0.
- Linear Regression (LR): We used the built-in function lm in R for implementing linear regression.
- Feedforward Neural Network (FNN): The Feedforward Neural Network model was implemented using the neuralnet library. We set the hidden layers to 7 and each with 5 neurons, respectively, and the linear output was set to TRUE. The key default parameters include an error threshold (threshold) of 0.01 and a maximum iteration step count (stepmax) of 1e+05.
- Autoregressive integrated moving average, ARIMA (ARI): The ARIMA model was automatically selected using the auto.arima function from the forecast package, with the input data being the length of the train dataset. By default, the function considers both non-seasonal and seasonal differences, with a maximum combined order of p+q+d of 5, and uses a stepwise selection approach.
- prophet (PRO): We implemented prophet using the prophet library. By default, it detects yearly and weekly seasonality and automatically identifies potential trend change points.
- K-Nearest Neighbors (KNN): The forecasting was performed using the knn_forecasting function. The model considered the previous 30 time points as lag variables, set by lags = 1:30. The number of nearest neighbors considered for the forecast was 50, as indicated by k = 50. The method used for forecasting was multi-input multi-output, specified by msas = “MIMO.”
- B-spline (B_S): The B-spline basis functions were generated using the bs function from the splines package. The degrees of freedom for the B-splines were set to values in the vector n0, which includes 5, 10, 15, and 20. The linear model was then fitted using the lm function between the response variable and the B-spline coefficients.
- Cubic plines (C_S): The ns function from the splines package was employed to generate natural spline basis functions. The degrees of freedom for the splines were specified by the vector n0, which includes the values 5, 10, 15, and 20. These degrees of freedom determine the complexity of the spline and the placement of knots in the data. The lm function was used to fit the model with y as the response variable and the spline coefficients as the explanatory variables.

## Supplementary Material

btaf508_Supplementary_Data

## Data Availability

All the SARS-COV-2 sequence data was available from GISAID. The processed historical mutation frequency data could be shared upon request.
