# Supplementary material for: Predicting the trend of SARS-CoV-2 mutation frequencies using historical data
Source: Bioinformatics. 2025 Sep 17;41(10):btaf508. doi: 10.1093/bioinformatics/btaf508 (PMC12502910; doi:10.1093/bioinformatics/btaf508)
Supplement: btaf508_Supplementary_Data [file btaf508_supplementary_data.pdf]

Supplementary Information

Supplemental Figures

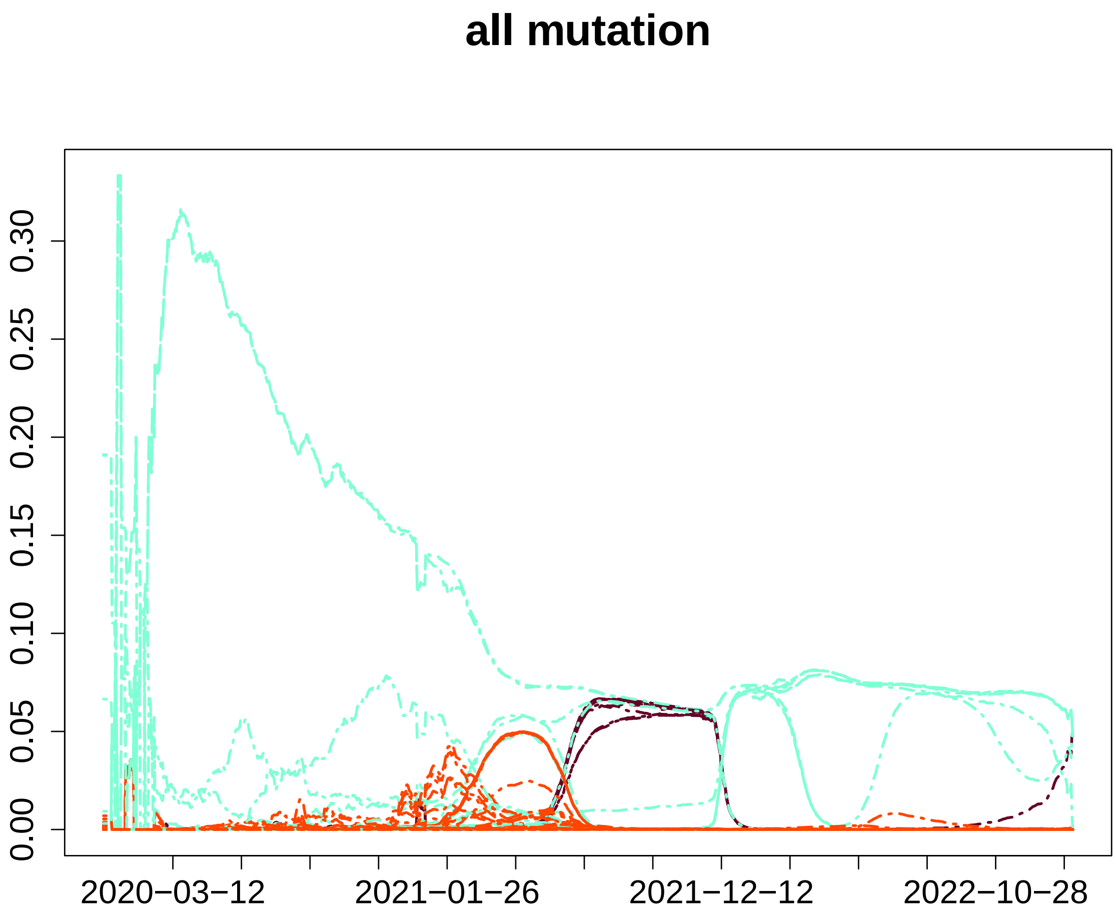

Figure S1: Mutation frequencies of all 87 mutation types, placed into three major categories, colored by cyan, orange red and maroon red. x-axis shows time, and the y-axis shows (smoothed) daily mutation frequencies.

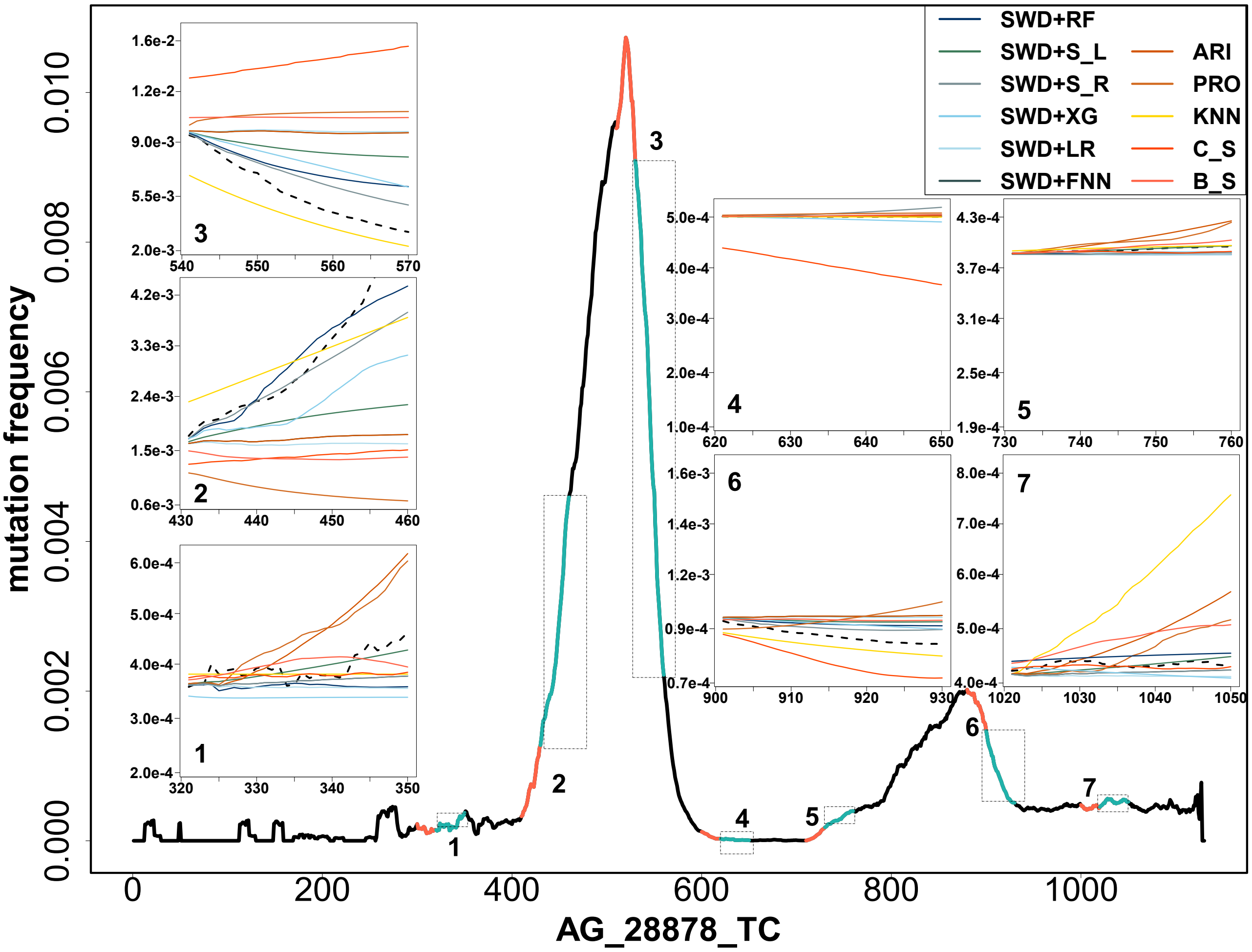

Figure S2: **Prediction performance on seven 30-day future segments of "AG_28878_TC"**. Here on the smoothed mutation frequency sequence of "AG_28878_TC" (black line) collected between 12/12/2019 to 1/26/2023, we selected seven distinct temporal locations, each comprising a 20-day input segment (marked in red) used for forecasting, followed by a 30-day ground truth segment (marked in cyan) used for evaluation. For each cyan segment, predictions from 11 different algorithms are shown. For each cyan segment, we demonstrated the prediction performance of the 11 different algorithms on predicting future 30 days. In each panel figure, the x-axis denotes the days, and y-axis the predicted or true mutation frequency.

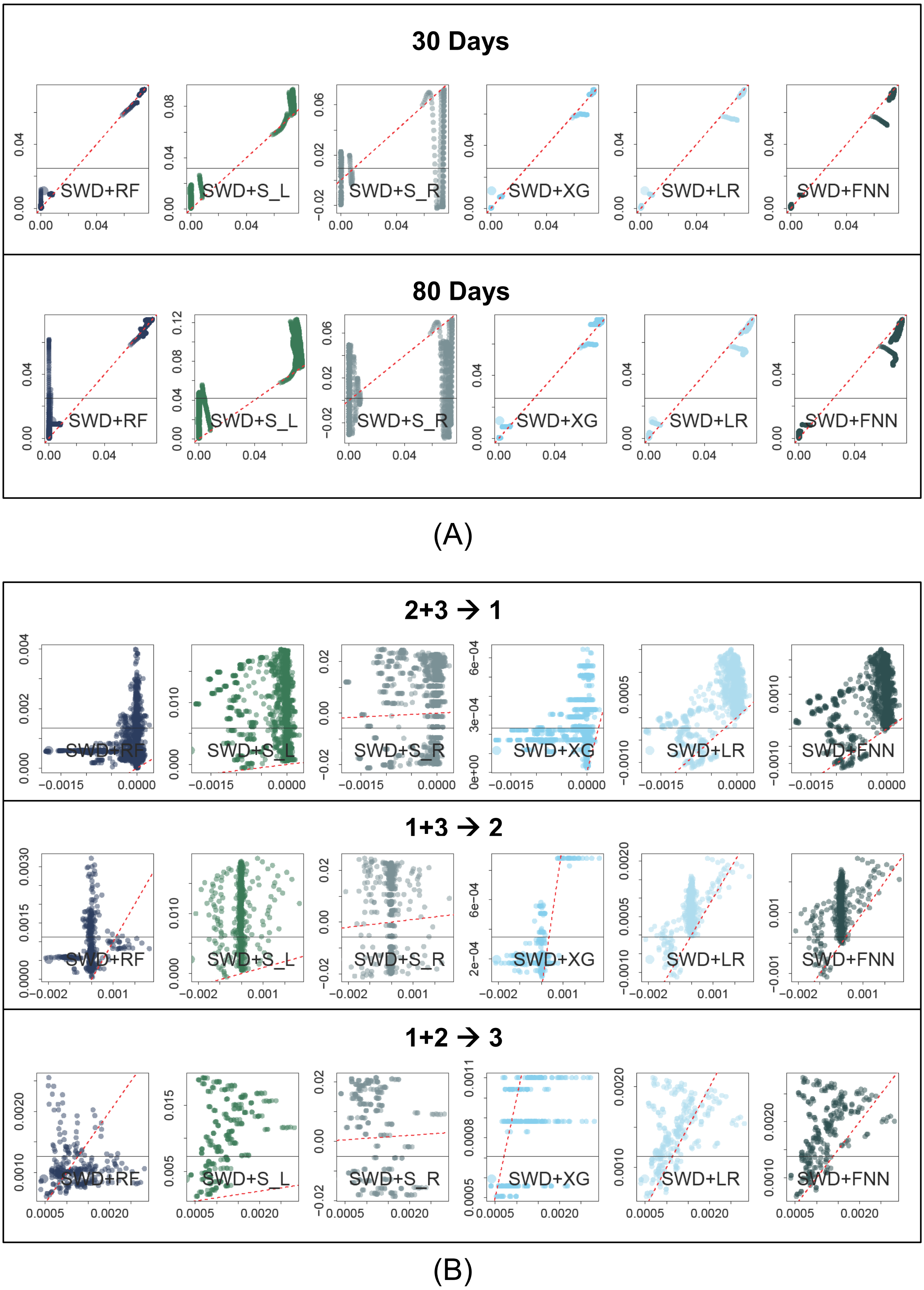

Figure S3: Prediction results using raw frequency data. (A) Scatter plot of observed (y-axis) and predicted (x-axis) mutation rate using raw frequency data for the next 30 (top) and 80 (bottom) days by gathering data of all mutations, similar to **Figure 3A, 3C**. (B) Robustness analysis for predicting the next 30 days by using two of the patterns as training and the rest as testing similar to **Figure 6**.
